# Fecal Microbiota Transplantation Inhibits Multidrug-Resistant Gut Pathogens: Preliminary Report Performed in an Immunocompromised Host

**DOI:** 10.1007/s00005-016-0387-9

**Published:** 2016-03-09

**Authors:** Jarosław Biliński, Paweł Grzesiowski, Jacek Muszyński, Marta Wróblewska, Krzysztof Mądry, Katarzyna Robak, Tomasz Dzieciątkowski, Wiesław Wiktor-Jedrzejczak, Grzegorz W. Basak

**Affiliations:** Department of Hematology, Oncology and Internal Diseases, Medical University of Warsaw, Banacha 1A, 02-097 Warsaw, Poland; Foundation for the Infection Prevention Institute, Warsaw, Poland; Department of Gastroenterology and Metabolic Diseases, Medical University of Warsaw, Warsaw, Poland; Department of Microbiology, Central Clinical Hospital, Medical University of Warsaw, Warsaw, Poland; Department of Dental Microbiology, Medical University of Warsaw, Warsaw, Poland; Department of Medical Microbiology, Medical University of Warsaw, Warsaw, Poland

**Keywords:** Fecal microbiota transplantation, Antibiotic-resistant bacteria, Gut colonization

## Abstract

Colonization of the gastrointestinal tract with multidrug-resistant (MDR) bacteria is a consequence of gut dysbiosis. We describe the successful utilization of fecal microbiota transplantation to inhibit *Klebsiella pneumoniae* MBL^+^ and *Escherichia coli* ESBL^+^ gut colonization in the immunocompromised host as a novel tool in the battle against MDR microorganisms.

*ClinicalTrials.gov identifier* NCT02461199.

Immunocompromised patients, particularly undergoing chemotherapy and prophylactic antibiotic treatment, are among those with poor gut microbiome repertoire, which promotes colonization by multidrug-resistant bacteria (MDR) (Ferrer et al. 2013; Montassier et al. 2015; Taur et al. 2012; Zhao et al. 2014; Zhang et al. 2013). The problem of antibiotic-resistant bacteria is growing worldwide and is caused by increased use of broad-spectrum antibiotics (Bell et al. 2014; ECDC/EFSA/EMA 2015; Spellberg et al. 2011). The field of hematology is especially affected because of frequent antibiotic prophylaxis and treatment due to severe immune suppression, caused by the disease and its treatment (Mikulska et al. 2014). Recent studies have revealed that human gut microbiota constitute a large reservoir of antibiotic resistance genes (Sommer and Dantas 2011; van Schaik 2015). Forslund et al. (2013) found resistance genes for 50 of 68 classes of antibiotics in 252 fecal metagenomes, at an average of 21 antibiotic resistance genes per sample. The gut reservoir for MDR bacteria includes naturally resistant ones (“resident resistome”) and those with acquired resistance (“variable resistome”) (Ruppé and Andremont 2013; Sommer et al. 2009; Sommer and Dantas 2011). The resistance genes are expressed also by bacteria not inhabiting the gastrointestinal (GI) tract in physiological conditions (Wellington et al. 2013). They may survive and colonize the GI tract for a long-term period. The density of colonizing MDR microorganisms may be as high as 10^9^/g of the stool and thus the colonized patient poses an epidemiological threat to other hospitalized individuals and household contacts (Ubeda et al. 2013). Carriage of antibiotic-resistant microbes in the GI tract is a risk factor of life-threatening systemic infection, especially during episodes of neutropenia and damage to the GI system, which enables translocation of pathogens into the bloodstream (Biliński et al. 2014; Taur and Pamer 2013). The composition of the intestinal microbiome itself determines so-called “colonization resistance”—protection against gut colonization by MDR organisms (Ubeda et al. 2013), while decreasing gut microbiome variability increases susceptibility to colonization by pathogenic bacteria.

Antibiotic treatment causes dysbiosis, which means quantitative, qualitative, metabolic, or locational imbalance of gut commensals (Hill et al. 2010). This is most likely caused not only by bactericidal effect of antibiotics, but also by changes in the interactions between flora and host intestinal cells: decreased production of IL-17, IFN-γ, decreased T cells stimulation promoting further imbalance (Candon et al. 2015). Therapeutic reversal of gut dysbiosis is more and more frequently used in clinical practice of fecal microbiota transplantation (FMT) for the treatment of relapsing *Clostridium difficile* infection. The strategy of FMT was shown to induce complete remission in up to 90 % of patients with *C. difficile* colitis (Austin et al. 2014), including these immunocompromised (Kelly et al. 2014) and has recently become a standard of care in relapsed and refractory patients (Debast et al. 2014).

It has been recently revealed in the murine model that reintroduction of healthy gut microbiota may lead to the eradication of vancomycin-resistant enterococci (VRE) colonizing the gut. Based on the similar mechanisms as mentioned above it is likely that a single particular strain can displace the pathogenic one (Ubeda et al. 2013). We hypothesized that FMT in humans may also be useful in eradication of the Gram-negative bacteria colonizing the gut. Here, we describe the first case of patient colonized with *Klebsiella pneumoniae* MBL (NDM, *NewDelhi metallo*-*β*-*lactamase*)^+^ and *Escherichia coli* ESBL^+^ who underwent FMT in our institution in order to decolonize/eradicate these bacteria from the GI tract.

A 51-year-old patient suffering from progressive multiple myeloma and having severely impaired both innate (absolute neutrophil count <1.0 × 10^9^/L) and acquired immunity (serum IgG concentration 3.83 g/L), treated in the past with multiple courses of chemotherapy including thalidomide, bortezomib and three autologus stem cell transplantations was identified as colonized in the gut with *K. pneumoniae* MBL (NDM^+^) and *E. coli* ESBL^+^. It was documented by repeated microbiological testing (five cultures over the last 6 months) including last test performed 1 week before FMT. At the time of FMT he was receiving lenalidomide and dexamethasone. The colonization status was documented by cultures of rectal swabs, which were done using standard microbiological techniques. Detection of extended spectrum beta-lactamases (ESBL) in Gram-negative rods was performed by a phenotypic method. The ability of isolates to produce carbapenemases was detected by phenotypic methods (MBL, KPC, OXA-48), Rapidec Carba NP biochemical assay (bioMerieux, France) and/or by Gene-Xpert qualitative real-time PCR (qPCR) method (Cepheid, USA).

Such patients (infiltration of the bone marrow, impairment of production of functional immunoglobulins, lenalidomide treatment) are at very high risk of developing systemic infection with MDR bacteria colonizing the gut. Though all the potential risks of FMT (e.g. transfer of unknown infections, prion diseases, impact on metabolism) are not known, we hypothesized that such infection poses greater risk to their life than the risk of the FMT. Therapeutic attempt to use FMT in this patient was reviewed and accepted by the Bioethical Committee of the Medical University of Warsaw and patient signed informed consent.

The fecal microbiota transplant was obtained from the stool of a 21-year-old, healthy, non-obese (BMI: 21) unrelated female donor who underwent thorough clinical examination and antimicrobial testing—negative results of: anti-HAV IgM and IgG, HBsAg, anti-HBc, HBV DNA; anti-HCV, HCV RNA, anti-HIV, HIV RNA, syphilis (serology), anti-CMV IgM and IgG, anti-EBV IgM and IgG, stool examination for parasites, GDH antigen and toxin A/B of *C. difficile* (EIA/ELISA or equivalent), enteropathogenic flora (classical culture). Based on family history, symptoms and clinical examination, the donor did not suffer from any autoimmune or metabolic diseases.

After obtaining informed consent, the day before FMT procedure, all prophylactic antibiotics (penicillin V, co-trimoxazole) were discontinued and the bowel lavage was performed with the oral laxative drug containing macrogols and sodium sulfate. The patient was fasting for at least 12 h and treatment with a proton pump inhibitor was introduced twice daily to neutralize gastric acid. The following day (26 February 2015), the fresh stool sample provided by the donor was processed in the laboratory according to a predetermined procedure. Over the next 2 h, approximately 100 g of stool was blended with 100 mL sterile physiological saline, passed three times through metal sieves to remove particulate material under sterile conditions and such material was infused to the patient’s small intestine via naso-duodenal tube. The course of treatment was uneventful and there was no inflammatory response. One hour after infusion, the patient passed one loose stool and felt a transient mild abdominal discomfort in the abdomen. He was discharged the next day. The control bacterial cultures from rectal swabs collected on day 10 and 26 after FMT repeatedly did not show growth of either *K. pneumoniae* NDM^+^ or *E. coli* ESBL^+^. In addition, the last stool sample was evaluated by Gene-Xpert qPCR (Cepheid, USA) searching for genes encoding carbapenemases: *bla*_KPC_, *bla*_NDM_, *bla*_VIM_, *bla*_IMP-1_, *bla*_OXA-48_. The test result was still positive for *bla*_NDM_ gene, as identified previously for colonizing *K. pneumoniae*. Due to advanced stage of the primary disease, the patient continued treatment with lenalidomide, occasionally receiving antibiotic prophylaxis for persistent neutropenia. He did not change his diet and did not use probiotics. For the first months following FMT he reported no infections, reduced severity of constipation and improved mood. The last evaluation of colonization status was planned at 6 months following FMT, but the patient was lost to follow-up shortly after microbiology assessment at 1 month.

Based on the assessments on day 10 and 26 after the procedure, the FMT may reduce carriage of bacteria below the threshold of culture of previously identified resistant *E. coli* and *K. pneumoniae*. Although the exact mechanism of this phenomenon is not known, one of the possible mechanisms was shown recently by Ubeda et al. (2013). Based on murine model of gut colonization with VRE, they revealed that eradication may be associated with direct inhibition of VRE by single component of healthy gut microbiota belonging to *Barnesiella* species. It is still unknown whether this effect is long lasting and whether its maintenance requires repetition of the procedure. Unexpectedly, the result of qPCR test for *bla*_NDM-1_ gene was shown to be positive, which might suggest that some other bacterial species carrying this gene still persist in the gut or that decolonization was not complete. Nevertheless, it is clear that FMT decreased the titre of colonizing microorganisms to levels not detectable by standard microbiological culture assay. Moreover, the result of the qPCR test must be interpreted with caution, because its very high sensitivity may lead to the detection of residual bacterial DNA in the gut, not necessarily coming from living microorganisms. It is noticeable that real-time PCR test has not been validated for this purposes in multicenter trials so far. Repeating culture and PCR testing at 6 months should dispel doubts. However, in our opinion, reduction of the titre of colonizing bacteria accomplishes the goal of the treatment, decreasing the risk of bacterial translocation through the wall of the gut into the bloodstream, as well as spread of these bacteria to other susceptible patients.

There have been few descriptions of FMT intentionally used to eradicate colonization with antibiotic-resistant bacteria from the GI tract (Crum-Cianflone et al. 2015; Freedman and Eppes 2014; Lagier et al. 2015; Singh et al. 2014) and our report confirms its utility. More systematic evaluation of efficacy of this treatment modality requires a prospective study, which is currently conducted in our institution.
